# PET/CT Scanner and Bone Marrow Biopsy in Detection of Bone Marrow Involvement in Diffuse Large B-Cell Lymphoma

**DOI:** 10.1371/journal.pone.0170299

**Published:** 2017-01-18

**Authors:** Fadi El Karak, Ibrahim R. Bou-Orm, Marwan Ghosn, Joseph Kattan, Fadi Farhat, Toni Ibrahim, Mario Jreige, Jean El Cheikh, Mohamad Haidar

**Affiliations:** 1 School of Medicine, Saint Joseph University (USJ), Beirut, Lebanon; 2 Nuclear Medicine Department, Centre Hospitalier Universitaire Vaudois (CHUV), Lausanne, Switzerland; 3 Hematology Department, American University of Beirut Medical Center (AUBMC), Beirut, Lebanon; 4 Radiology Department, American University of Beirut Medical Center (AUBMC), Beirut, Lebanon & Nuclear Medicine Department, Mount Lebanon hospital, Beirut, Lebanon; Northwestern University Feinberg School of Medicine, UNITED STATES

## Abstract

Evaluation of bone marrow involvement (BMI) is paramount in diffuse large B-cell lymphoma (DLBCL) for prognostic and therapeutic reasons. PET/CT scanner (PET) is now a routine examination for the staging of DLBCL with prognostic and therapeutic implications. This study evaluates the role of PET for detecting marrow involvement compared to bone marrow biopsy (BMB). This monocentric study included 54 patients diagnosed with DLBCL between 2009 and 2013 and who had FDG PET/CT in a pre-treatment setting. A correlation analysis of the detection of BMI by PET and BMB was performed. A prognostic evaluation of BMI by BMB and/or PET/CT and correlation with an overall 2-year survival were analyzed. PET was more sensitive for the detection of BMI than BMB (92.3% vs. 38.5%). It can be considered a discriminatory Pre-BMB test with a negative predictive value of 97.6%. In addition, BMI by PET had a prognostic value with strong correlation with progression-free survival (PFS) (HR = 3.81; p = 0.013) and overall survival (OS) (HR = 4.12; p = 0.03) while the BMB had not. PET shows superior performance to the BMB for the detection of marrow involvement in DLBCL. It may be considered as the first line examination of bone marrow instead of the biopsy.

## Introduction

Diffuse large B cell lymphoma (DLBCL) is the most common Non-Hodgkin Lymphoma, representing nearly 30% of all Non- Hodgkin Lymphoma in Lebanon [1]. The evaluation of bone marrow involvement (BMI) is paramount in the case of DLBCL for prognostic and therapeutic reasons [2]. The current recommendations, such as those of the European Society of Medical Oncology, insist on bone marrow biopsy (BMB) in the initial evaluation of all patients diagnosed with DLBCL [3].

This intervention remains an invasive and painful procedure with a significant number of complications [4]. Although the PET/CT scanner (PET) is now a routine procedure for the staging of DLBCL with prognostic and therapeutic implications as well as for post-treatment assessment, its role in BMI as a non-invasive examination remains a current debate in oncology clinic. The aim of this study is to evaluate the correlation between PET and BMB in detecting BMI in patients with DLBCL. A secondary objective is to assess the prognostic value of BMI detected by PET on patients’ survival with an ultimate goal of verifying the eligibility of some hypotheses found in the literature.

## Methods

### Study design

This study is a retrospective study performed in a single institution in Beirut- LEBANON. All patients diagnosed with DLBCL between 2009 and 2013, who have undergone a pre-therapeutic PET and BMB were included into the analysis. A total of 54 patients were identified.

### Variables

In addition to sociodemographic variables such as age and gender, data were collected on the clinical characteristics of cases. Variables under this section were: staging, International Prognostic Index (IPI), the presence of B symptoms (fever, weight loss, night sweats) as well as the the presence of bulky disease, splenic and extra nodal involvement. All these variables were collected from the hospital medical records. Moreover, bone marrow involvement (BMI) was considered as the main independent variable that was blindly assessed based on PET and BMB. Only one PET scan machine was used in the staging of DLBCL lymphoma and the results were blindly read twice independently from the course of the disease in the same radiology department by at least two “nuclear medicine specialists” with more than 15 years of experience in PET/CT scan: the first reading in the period of disease staging (information were collected from the PET reports signed by two different physicians) and the second one during this study to verify the findings. None of the PET scan results was found to be inaccurate during the proofreading. Positive lesion on PET scan was defined as a focal increased uptake or diffuse increased heterogeneous bone marrow uptake greater than liver uptake with a mean SUV max of more than 3.8. As for the bone marrow biopsy, the results were read only in the staging period in an accredited pathology laboratory that follows international guidelines and has decades of experience. The study used the pathology reports and did not verify retrospectively the results since pathology findings are not currently debated and are already valid. All the included biopsies were adequate since they had a bone marrow core with a size of more than 0.5 cm.

### Statistical analysis

Two analysis were conducted for the sensitivity/specificity of the tests to investigate a potential difference between the current recommendations of assessing the bone marrow involvement by the biopsy and an eventual role of PET scan in this assessment. As for the prognostic value of the two exams, the log-rank test was used in the survival analysis and Hazard Ratios (HR) were reported to compare between sub-groups. The statistical analyses were tested with PASW (Version 18) software.

### Ethical considerations

The study was in accordance with the ethical standards of the institutional research committee and with the Helsinki declaration. For this type of study, formal consent is not required.

## Results

### Patients characteristics

#### Demographic and clinical data

Among the 54 patients diagnosed with DLBCL, 29 (54%) were men. Mean age was 50 years (16–87).

The majority of patients had stage IV disease at diagnosis (41%), while stages I, II and III were diagnosed in 18.5%, 22% and 18.5% respectively. Bulky disease, splenic involvement and extranodal disease were present in 30%, 7% and 48% of patients respectively. All patients were treated with R-CHOP based regimen. Patients’ characteristics are detailed in Table 1.

**Table 1:** Demographic and clinical patient characteristics.

Gender	Male, n (%)	25 (46)
	Female, n (%)	29 (54)
Age	Mean ± SD, years Range, years	50±19 16–87
Stage	I, n (%)	10 (18.5)
	II, n (%)	12 (22)
	III, n (%)	10 (18.5)
	IV, n (%)	22 (41)
B Symptoms, n (%)		7 (13)
Bulky disease, n (%)		16 (29)
Splenic disease, n (%)		4 (7)
Extranodal disease, n (%)		26 (48)
International Prognostic Index (IPI)	Mean± SD Range	2±1 0–4

#### Survival analysis

After a mean follow up of 30-months, 12 out of 48 patients (25%) developed a relapse of their disease and 8 died. The 1- and 2- year progression-free survival (PFS) were 77% and 1- and 2- years overall survival (OS) were 95% and 84% respectively.

#### Features of BMI

PET detected BMI in 12 patients (22%) with only 1 patient showing diffuse infiltration. BMB detected BMI in 5 patients only (9%); 4 of which having also marrow involvement on PET. 41 patients had a negative BMB and a negative PET exam. Concordance rate between PET and BMB is 83.3% (45 of 54 patients). Table 2 shows the characteristics of marrow involvement detected by PET and/or BMB.

**Table 2:** Characteristics of bone marrow involvement.

Characteristics of bone marrow involvement on PET and BMB	n (%)
PET + / BMB -	8 (15)
PET + / BMB +	4 (7)
PET − / BMB +	1 (2)
PET − / BMB -	41 (76)
Total	54

### Correlation analysis of the detection of BMI by PET and BMB

If we consider BMB as the gold standard and the only true positive procedure for detecting BMI, PET showed sensitivity and specificity of 80%. The negative predictive value of this test would be 98% and the positive predictive value would be 33%.

If we consider any BMI as a true positive whether detected by BMB or PET, PET sensitivity would be of 92% (12/13) vs. 38% (5/13) for the BMB. The specificity would be of 100% for both exams.

An assessment of the concordance between the PET and BMB results was also performed using Receiver Operating Characteristic curve. Analysis showed weak but significant concordance between PET and BMB (kappa = 0.391 < 0.6 with p = 0.001). The discriminative power of PET for BMI when considering BMB as gold standard exam was 0.82 > 0.5 (p = 0.02).

### Prognostic values of BMB and PET and correlation with the 2-year survival rate

#### Prognostic value of the BMB and PET on PFS

Mean PFS of patients with positive and negative BMI by BMB was identical. The Hazard Ratio (HR) for relapse according to BMB was 0.61 (CI 95% [0.13–2.78]; p = 0.517).

**Fig 1:**
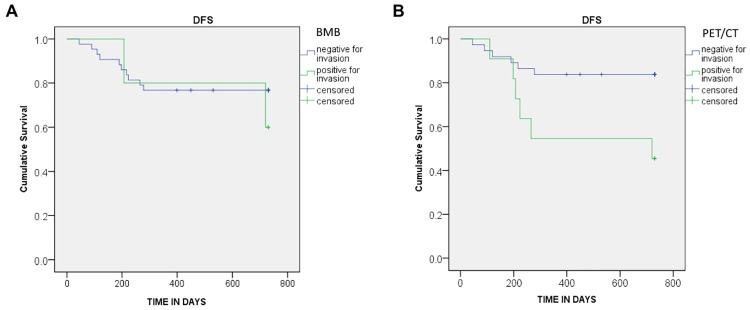
PFS curves. The PFS curves are provided according to the result of bone marrow involvement (a) BMB results (HR: 0.61 [0.13–2.78]; p = 0.517) (b) PET results (HR: 3.81 [1.23–11.87]; p = 0.013).

There was a significant difference for disease relapse between patients with positive and negative BMI detected by PET. The mean PFS of patients with or without BMI on PET was 16.2 and 21.2 months respectively. HR was 3.81, CI 95% [1.23–11.87] and p = 0.013 (Fig 1).

#### Prognostic value of the BMB and PET on OS

Mean OS of patients with positive and negative BMI by BMB was identical. HR for death according to BMB was 0.83 (CI 95% [0.10–6.77]; p = 0.864).

**Fig 2:**
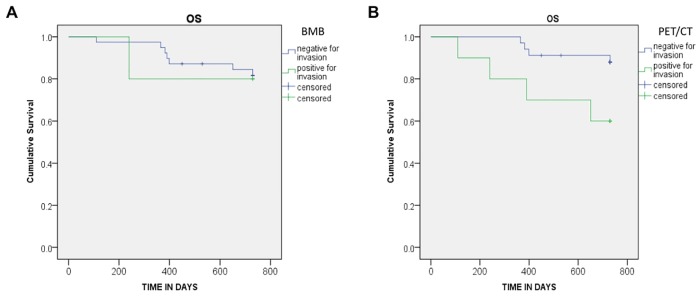
OS curves. OS curves are provided according to the result of bone marrow involvement. (a) BMB results (HR: 0.83 [0.10–6.77]; p = 0.864) (b) PET results (HR: 4.12 [1.03–16.5]; p = 0.03).

There was a significant difference for the risk of death between patients with positive and negative BMI detected by PET. The mean OS of patients with or without BMI on PET were 19.2 and 23.3 months respectively. HR was 4.12, CI 95% [1.03–16.5] and p = 0.03 (Fig 2).

## Discussion

This retrospective study aims to compare the accuracy of lymphoma BMI detection by BMB and PET. A specialized nuclear radiologist reviewed PET imaging of 54 patients with DLBCL who have a PET scanner and BMB performed before therapy. Our study showed discordance between the two examinations with PET showing an excellent sensitivity (92%) and a very high negative predictive value (98%) when compared to BMB. BMI by PET had a negative impact on PFS and OS while BMB had no predictive effect.

Our results are concordant with other trials that questioned the value of the BMB as a single examination for assessing BMI in the NHL and especially DLBCL. Our mismatch rate of 17% (9 of 54 patients) between PET and BMB in the detection of BMI is similar to the one found in the study of Hong et al [5] where the rate was 19%. Many explanations for this discrepancy were postulated, such as the small size of the sample obtained during a “blind” biopsy [6] and the focal disease of bone marrow involvement, away from the iliac crest that represents 92% (11 of 12 patients) of cases of marrow involvement detected by PET in our series. There is no current debate in the literature about the quality of the biopsy. It is not the reason behind re-considering the use of BMB in patients with DLBCL. Its invasive nature and its limitation to the assessment of one region in the body are the main problems that pushed scientists to evaluate other alternatives such as PET/CT scan.

Our results are also in line with the meta- analysis reported by Adams et al [8] with a high PET sensitivity (92%) and high negative predictive value (98%).

**Fig 3:**
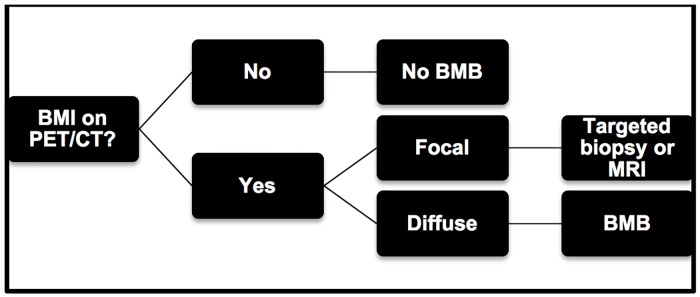
BMI evaluation. Proposed algorithm for the evaluation of Bone Marrow Involvement (BMI) in DLBCL patients.

The discriminative power of PET for BMI when considering BMB as gold standard exam using Receiver Operating Characteristic was 0.82 > 0.5 (p = 0.02) which indicates that PET can be considered a good discriminatory pre-BMB. These results allow us to suggest that PET could be a first-line evaluation tool for BMI in DLBCL patients: a negative result cancels the indication of the BMB while a positive result shows an indication for a targeted biopsy or MRI in focal involvement and a blind bone marrow biopsy in diffuse disease (Fig 3).

By excluding the systematic BMB from the baseline work-up of newly diagnosed DLBCL and considering the PET as the only method to assess the bone marrow, an estimation of 3% of cases having bone marrow involvement would be unrevealed. [7]. However, this would not result in any modification in patients’ therapeutic plan [8,9].

Although this study had a relatively small sample size, it generated data on a potential prognostic value of BMI revealed by PET. It showed that a PET exam showing a positive BMI was associated to more frequent events of disease relapse and death at 2 years with HR 3.81 and 4.12 respectively. This result was also found by Berthet et al [10]. The study compared the PFS and OS at 2 years in 133 patients according to bone marrow invasion by BMB and PET. Their study showed that bone marrow disease on PET was an independent prognostic factor for PFS and OS. However, this result was also true for BMB, which contradicts our results.

Three other studies have evaluated the effect of BMI by PET and BMB on the disease prognosis. Adams et al [11] in a series of 78 patients reported that BMI on PET had no prognostic value. Only BMI by BMB was an independent predictive factor and it has even exceeded the effect of IPI score according to their study. The lack of significant difference in PFS and OS according to BMI on PET was also noticed by the series of Hong et al. In addition, Khan et al [8] assessed the survival of 44 patients with stage IV and found that patients with BMI on PET had similar PFS and OS to patients with stage IV without BMI; however, the positivity of BMB had a pejorative additional effect in the population studied.

Our study population was homogenous and a retrospective review of PET was performed for BMI re-assessment. Retrospective analysis with a mean of 30 months was sufficient to correlate results of BMB and PET with the survival of patients, a new concept not well studied so far in the literature. Our study aimed to establish a new hypothetical recommendation for patients with DLBCL to find an alternative to an invasive and painful technique.

The study has several limitations. The retrospective nature of the study prevented us to expand the patients monitoring. The study also failed to realize targeted bone marrow biopsy for focal involvement detected on PET for histological confirmation, which was also seen in other studies. Finally, the number of patients included in all studies including ours, is a major limitation regarding the generalization of the results; therefore, more robust prospective studies are needed to assess if a BMI detected by PET according to multiple nuclear specialists could have an independent prognostic value on patients’ survival. Future research should also evaluate the effects of each type of BMI on PET (focal and diffuse) independently after adjusting for other predictors of patients’ survival.

The study, although retrospective, concluded that PET read by specialists has an excellent sensitivity for detecting BMI in DLBCL. This sensitivity is superior to BMB especially in the case of focal disease and sparing of the iliac crests. The BMI detected by PET has also showed in our series a prognostic value correlated with patient survival. Blind BMB might be unnecessary in the era of nuclear imaging; hence, the need to review its systematic indication in staging of DLBCL. We suggest a theoretical algorithm for the evaluation of BMI in DLBCL: a negative PET result for BMI should eliminate the need for BMB; only patients with BMI on PET will benefit from a targeted biopsy in focal involvement or a blind biopsy in case of diffuse involvement. This algorithm should be evaluated in a prospective study or revalidated in a large retrospective study to be possibly adopted in the recommendations for staging.

## Supporting Information

S1 Dataset Dataset of the sample of patients included in the study. (XLSX)
